# Effect of closed-loop vibration stimulation on sleep quality for poor sleepers

**DOI:** 10.3389/fnins.2024.1456237

**Published:** 2024-10-07

**Authors:** Hyun Bin Kwon, Jonghyeok Jeong, Byunghun Choi, Kwang Suk Park, Eun Yeon Joo, Heenam Yoon

**Affiliations:** ^1^Research Institute of BRLAB, Inc., Seoul, Republic of Korea; ^2^Department of Biomedical Engineering, College of Medicine, Seoul National University, Seoul, Republic of Korea; ^3^Department of Neurology, Samsung Medical Center, Sungkyunkwan University School of Medicine, Seoul, Republic of Korea; ^4^Department of Human-Centered Artificial Intelligence, Sangmyung University, Seoul, Republic of Korea

**Keywords:** sleep modulation, autonomic modulation, closed-loop stimulation, vibratory stimulation, synchronization, coherence, sleep quality

## Abstract

**Introduction:**

Recent studies have investigated the autonomic modulation method using closed-loop vibration stimulation (CLVS) as a novel strategy for enhancing sleep quality. This study aimed to explore the effects of CLVS on sleep quality, autonomic regulation, and brain activity in individuals with poor sleep quality.

**Methods:**

Twenty-seven participants with poor sleep quality (Pittsburgh sleep quality index >5) underwent two experimental sessions using polysomnography and a questionnaire, one with CLVS (STIM) and the other without (SHAM).

**Results:**

Sleep macrostructure analysis first showed that CLVS significantly reduced the total time, proportion, and average duration of waking after sleep onset. These beneficial effects were paralleled by significantly increased self-reported sleep quality. Moreover, there was a significant increase in the normalized high-frequency (nHF) and electroencephalography relative powers of delta activity during N3 sleep under STIM. Additionally, coherence analysis between nHF and delta activity revealed strengthened coupling between cortical and cardiac oscillations.

**Discussion:**

This study demonstrated that CLVS significantly improves sleep quality in individuals with poor sleep quality by enhancing both subjective and objective measures. These findings suggest that CLVS has the potential to be a practical, noninvasive tool for enhancing sleep quality in individuals with sleep disturbances, offering an effective alternative to pharmacological treatments.

## 1 Introduction

Sleep is a fundamental aspect of daily life as it is crucial for growth, survival, and wellbeing by maintaining physiological homeostasis and re-energizing the brain and body (Dinges, 2014; Helakari et al., 2023). Chronic sleep deprivation can lead to numerous negative health outcomes, including increased risk of cardiovascular diseases, diabetes, obesity, and impaired immune function (Beckers et al., 2006; Mestivier et al., 1997). Moreover, poor sleep is associated with decreased cognitive performance and heightened emotional distress, contributing to diminished quality of life (Blaxton et al., 2017; Bonnet and Pettis Memorial, 1985). The critical role of sleep and increasing prevalence of sleep disorders have underscored the need for effective interventions to enhance sleep quality. Thus, various sleep modulation methods have been proposed to modulate sleep (Malkani and Zee, 2020; Park et al., 2023).

These methods need to be both effective and practical to improve sleep; thus, they must be noninvasive and nondisruptive. One such approach is vestibular stimulation, which involves the gentle rocking of vibrational movements. Bayer et al. (2011) used a continuous rocking stimulation (0.25 Hz) with a bed equipped with an electrical rotor to produce vibrational excursions in 12 good sleepers during naps. The results showed reduced sleep latency, increased non-rapid eye movement (non-REM) sleep, and enhanced sleep spindles. In a study applying the same methodology to nocturnal polysomnography (PSG) in 18 healthy young adults, an increase in the duration of the N3 sleep stage and a decrease in arousal were observed (Perrault et al., 2019). Additionally, there was an increase in the density of slow oscillation (SO) waves and sleep spindles during the N3 sleep stage, along with enhanced declarative memory consolidation.

Auditory stimulation is another technique used for sleep enhancement. Ngo et al. (2013) detected the ongoing rhythmic occurrence of SO up states in the night sleep of 11 healthy participants in real time and delivered a 50-ms duration of pink noise through earphones at a calibrated sound pressure level of 55 dB. As a result, auditory stimulation in the phase with SO upstate enhanced and extended the sequence of SOs during sleep and increased the retention rate of declarative memory. Another study showed that phase-locked auditory stimulation with slow waves (SWs) had similar benefits during an afternoon nap (Ong et al., 2016). The researchers applied blocks of five tones synchronized with SWs to 16 healthy young adults during a 90-min nap. The results showed increased SW amplitude, theta, and fast spindle activity and enhanced declarative memory retention. Debellemaniere et al. (2018) validated an ambulatory wireless dry electroencephalography (EEG) device designed for home use that enabled closed-loop auditory stimulation during sleep. This device automatically detected N3 sleep and delivered stimulating sounds through bone conduction to 20 healthy young participants. In experiments conducted in a home environment, they observed that auditory stimulation triggered by SO increased delta power by 43.9% and enhanced SO responses to auditory stimulation. Auditory stimulation triggers the extension of SOs and SWs and enhances declarative memory consolidation. However, there were no statistically significant differences in PSG-derived sleep architecture (Stanyer et al., 2022).

Heart rate can be entrained by weak external stimulation signals, such as sound, light pulses, and vibration (Anishchenko et al., 2000; Choi et al., 2019; Yoon et al., 2019). A study with 10 participants applied weak external vibration stimulation at a frequency approximately 3% lower than the spontaneous heart rate of each participant during nap sleep, followed by heart rate variability (HRV) analysis (Choi et al., 2019). The results showed an increase in the normalized high-frequency (nHF) parameter and a decrease in the normalized low-frequency (nLF) parameter, indicating the modulation effects of the closed-loop vibration stimulation (CLVS) on the autonomic nervous system (ANS) during sleep. The same methodology was applied to the nocturnal sleep of 12 participants while simultaneously performing PSG (Choi et al., 2021). These results, consistent with those of previous studies, showed an increase in the nHF parameter and a decrease in the nLF parameter during N3 sleep. Moreover, the power of SW activity and density of SO increased along with an increased retention ratio in the declarative memory test. There was a positive correlation (*r*^2^ = 0.752, *p*-value = 0.005) among the synchronization ratio of the participants’ heart rate, vibratory stimulation, and retention ratio. This suggests that weak external vibration stimulation promotes SWS stage depth and memory retention by shifting autonomic balance.

Autonomic modulation can be implemented as an unobtrusive heart rate monitoring method without attaching sensors (Kwon et al., 2021; Ravindran et al., 2023), making it one of the most practically applicable noninvasive stimulation methods for sleep modulation (Park et al., 2023). However, in a study by Choi et al. (2021), this method did not lead to changes in sleep structure, possibly because the participants were healthy individuals who already had good sleep quality, which made improvement difficult. Stress, depression, and fatigue, which are considered risk factors for sleep disturbances, lead to poor sleep quality associated with decreased parasympathetic modulation and increased sympathovagal balance during sleep (Burton et al., 2010; Hall et al., 2004; Kwon et al., 2019). Therefore, we hypothesized that the CLVS might have a facilitating effect on sleep improvement in populations exhibiting disturbed sleep.

This study aimed to investigate the effects of CLVS on sleep quality in individuals with poor sleep quality. Twenty-seven participants with poor sleep quality spent a night with CLVS and a night without CLVS, and we compared the sleep quality using PSG-derived parameters and self-report scales, cortical brain activity using EEG, and ANS activity using HRV across the two nights. We further investigated the coherence between the relative power of sleep EEG and HRV to assess the effect of CLVS on the interaction between the central nervous system (CNS) and ANS.

## 2 Materials and methods

### 2.1 Participants

The participants were recruited from Samsung Medical Center between August 1, 2023 and December 31, 2023. The study participants were recruited through offline advertisements. The inclusion criteria were as follows: (1) age 20–60 years and (2) poor sleep quality [Pittsburgh Sleep Quality Index (PSQI) > 5]. The exclusion criteria were (1) inability to complete the questionnaires owing to intellectual disability; (2) severe sleep apnea [apnea-hypopnea index (AHI) > 30]; (3) Periodic Limb Movement Index (PLMI) > 15; (4) narcolepsy, insomnia, or depression; and (5) neurological or cardiovascular disorders. These criteria were considered potential factors affecting the quality of ballistocardiogram (BCG) signals or autonomic control during sleep (Rauhala et al., 2009; Kwon et al., 2022). Initially, 31 participants were recruited through online and offline advertisements. However, data collection failed for two participants because of handling errors made by the researcher conducting the initial experiments, and two participants withdrew during the experiment. In total, 27 participants [13 men and 14 women; mean age, 44.4 years ±8.1 standard deviation (SD); mean body mass index, 23.2 kg/m^2^ ± 2.6 SD; and mean PSQI, 8.2 ± 2.1 SD] were included in this study.

All study procedures were performed in accordance with the 1964 Declaration of Helsinki, as revised in 2013. The study protocol was approved by the Institutional Review Board of Samsung Medical Center (protocol code 2023-08-176). All the participants provided informed consent.

### 2.2 Experimental design

**Figure 1:**
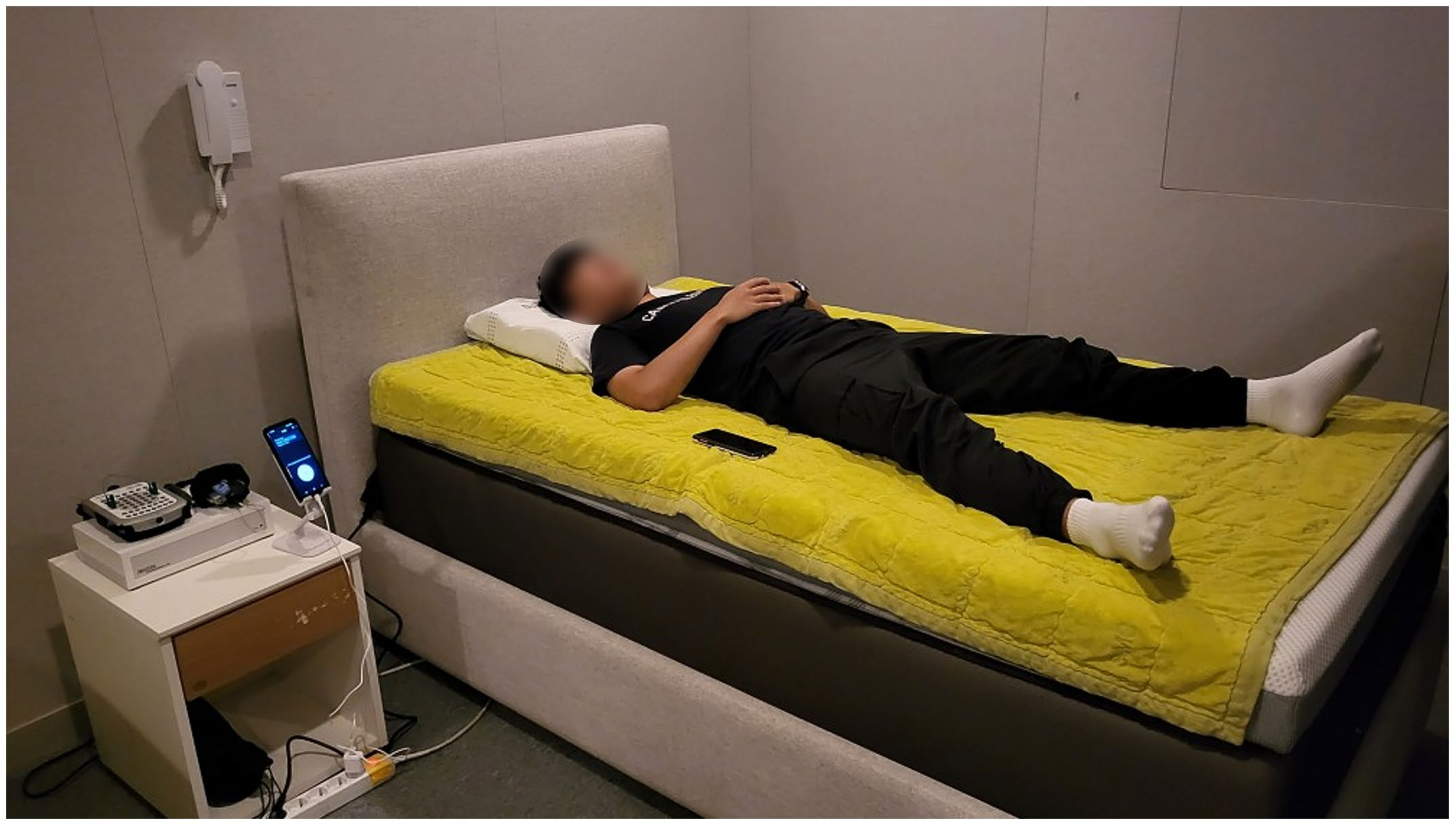
Installation of the smart mattress in the sleep laboratory with PSG in our experiment.

In this study, a smart mattress (Benzamin AI Sleep Controller M1; BRLAB Inc., Korea) was installed in a sleep laboratory at Samsung Medical Center (Figure 1). The participants visited the sleep laboratory twice, at least 1 week apart, on the same day of the week. One visit was designated as the STIM night, in which autonomic modulation was used, and the other visit was designated as the SHAM night, in which autonomic modulation was not used; thus, a blind test was conducted. The order of SHAM and STIM nights was randomly assigned, and the intensity of the vibration for autonomic modulation was set significantly weak that the participants could not perceive whether the stimulation was being applied. Additionally, to avoid differences in sleep duration compared with usual, the participants were prohibited from consuming caffeine on the day of the experiment. After waking up, all participants completed a questionnaire that included questions about their sleep quality the previous night and their perception of vibration stimulation during sleep.

PSG was recorded with Somnologica (Embla, Denver, CO, USA) using a six-channel EEG, four-channel electrooculogram, electromyogram, and electrocardiogram (ECG). A thermistor, nasal air pressure monitoring sensor, oximeter, piezoelectric band, and body position sensor were also attached to the participants. Sleep stages during 30-s epochs, arousals, and respiratory events were scored by professional technicians according to the American Academy of Sleep Medicine manual (Berry et al., 2017).

### 2.3 Smart mattress

**Figure 2:**
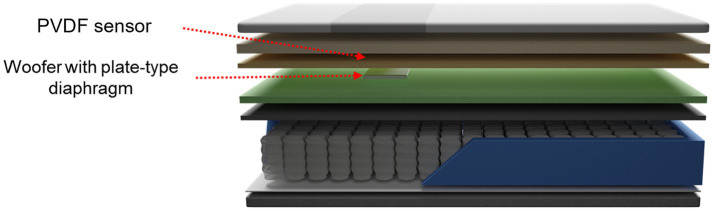
Schematic of the smart mattress used in the experiment.

The smart mattress is composed of an unconstrained sensor to measure the BCG, a woofer that provides vibrational stimulation, and memory foam (Figure 2). In this study, a standard mattress with dimensions of 110 cm width and 200 cm length was used. The embedded sensor is a polyvinylidene fluoride (PVDF) film sensor, which is an 80-cm-long strip with a thickness of approximately 30 μm. It was positioned 53 cm above the mattress and 4 cm from the cover. This placement is optimal for measuring heart rate and respiratory signals from the user’s chest. The woofer was located 2 cm below the PVDF film sensor and was attached to a plate-type diaphragm to facilitate efficient vibration transmission. Both the PVDF sensor and woofer were connected to a microprocessor that detected the individual’s heart rate and output vibration at preset levels.

**Figure 3:**
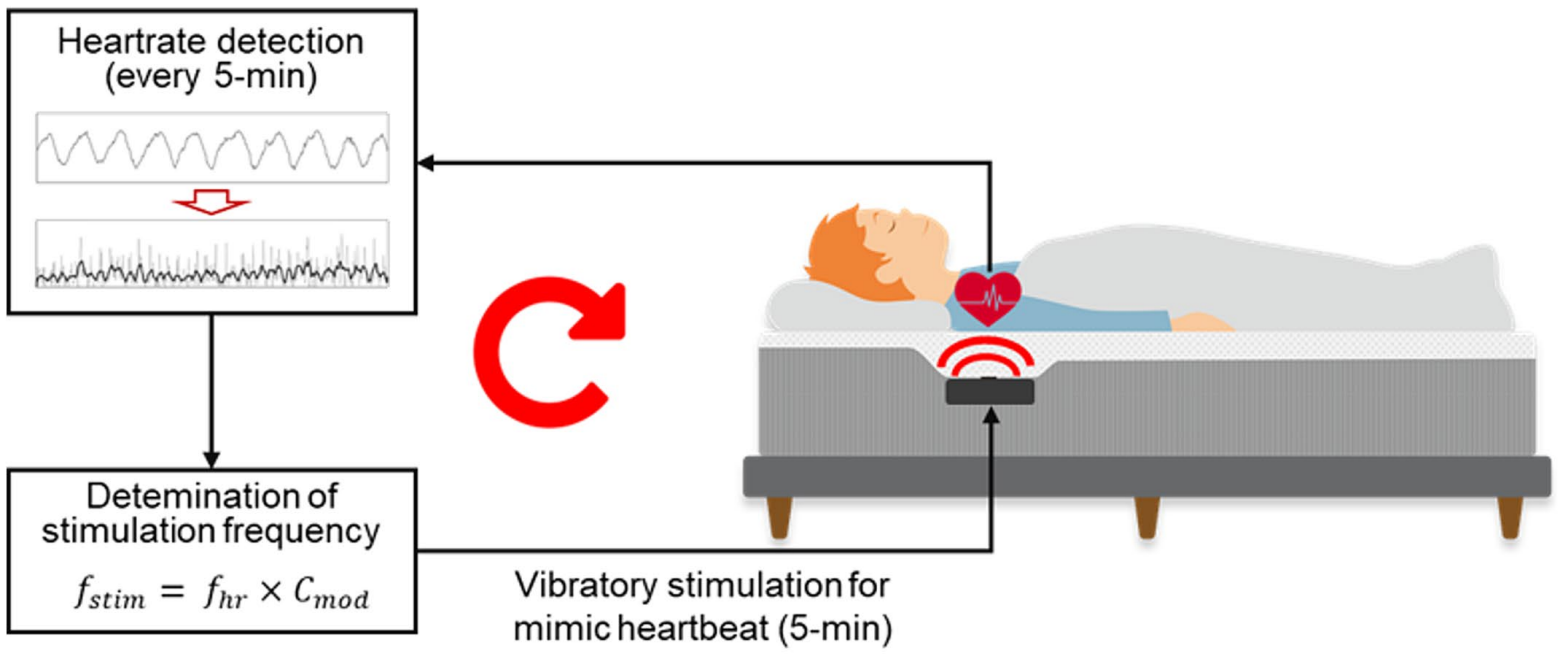
Diagram of the CLVS system embedded in the smart mattress.

The autonomic modulation function of the smart mattress is shown in Figure 3. The microprocessor used the signal processing method described by Choi et al. (2009) to automatically detect the J-peak in the BCG measured by the PVDF film sensor and calculate the average heart rate ( $f_{\mathrm{hr}}$ ) over the previous 5 min. The estimated 5-min average heart rate demonstrated high reliability, with a Pearson’s correlation coefficient of 0.903 and a mean absolute percentage error of 2.139%, when compared with the simultaneously measured heart rate calculated -based on ECG R-peak intervals (Supplementary Figure S1). The subsequent 5-min segment then output the heart rate-mimicking vibrations at a frequency multiplied by a specific coefficient ( $C_{\text{mod}}$ ). During the STIM night, stimulation was applied at −3% rate ( $C_{\text{mod}}$ = 0.97) of the average heart rate from the previous 5 min, as this rate has been the most effective in modulating the heart rhythm in previous studies (Choi et al., 2019, 2021). Therefore, vibrations were applied to the participants throughout the whole night, excluding the first 5 min after the PSG recording began. During the SHAM night, the average heart rate was calculated every 5 min; however, the vibrational stimulation was muted.

### 2.4 Heart rate variability and synchronization analysis

To investigate the effects of CLVS on the intrinsic heart rhythm during sleep, we analyzed HRV and heart rate synchronization with vibration stimuli across different sleep stages: N1 sleep, N2 sleep, N3 sleep, REM sleep, and wake after sleep onset (WASO). HRV, a method used to evaluate the ANS activity, was computed based on the interval between the R-peaks in the ECG signals. The R peaks were initially detected using an automated peak detection algorithm (Choi et al., 2019), and subsequently visually corrected and confirmed.

HRV time-domain parameters included mean heart rate and the SD of intervals between R-peaks. HRV frequency-domain parameters included nLF, nHF, total frequency (TF), and high frequency-low frequency ratio (HFLF). Frequency-domain parameters were calculated by computing the power within the 0.04–0.15 Hz (LF) and 0.15–0.4 Hz (HF) bands and normalizing by the TF power. The HFLF ratio was derived from the power ratio of HF to LF. Time-domain parameters were calculated every 30-s epoch, whereas frequency-domain parameters were computed using a sliding window of 10 epochs, shifting one epoch at a time. Segments with motion artifacts or ectopic heartbeats were excluded from the analysis. To account for individual differences, the HRV parameters were normalized to the values obtained during sleep onset latency (SOL) (Lee et al., 2024).

To analyze the synchronization between the participants’ heartbeats and vibratory stimulation, we followed a series of computational steps based on the methods outlined in Rosenblum et al.’s study (Rosenblum et al., 2001). We calculated the instantaneous phases of the R-peak times of the ECG signals and stimuli. The instantaneous phase $\phi_{i}\left(t\right)$ for each R-peak and stimuli was computed using the following linear interpolation between successive R-peaks, as described in Equation (1):


(1) $$\phi_{1}\left(t\right)=2\pi k+2\pi \frac{t-t_{k}}{t_{k+1}-t_{k}},t_{k}\leq t<t_{k+1}$$

where $t_{k}$ and $t_{k+1}$ are the times of consecutive R-peaks and k is the index of the R-peak. Next, the phase difference between the two signals was computed. Given phases $\phi_{1}\left(t\right)$ and $\phi_{2}\left(t\right)$ , the phase difference was determined as described in Equation (2):


(2) $$\varphi_{n,m}\left(t\right)=n\phi_{1}\left(t\right)-m\phi_{2}\left(t\right)$$

In this analysis, we set n and m as 1. To account for the cyclic nature of the phases, the phase difference was wrapped into the interval [0,2π] using the modulo operation, as described in Equation (3):


(3) $$\Psi_{n,m}=\varphi_{n,m}\;\mathrm{mod}\;2\pi$$

This cyclic relative phase $\Psi_{1,1}\left(t\right)$ was then used to analyze the synchronization. A histogram of the relative cyclic phases was constructed to observe their distribution. The probability density $p_{k}$ of finding $\Psi_{1,1}\left(t\right)$ within the 𝑘-th bin of the histogram was calculated. The Shannon entropy S of the cyclic relative phase distribution was calculated using the following Equation (4):


(4) $$S=-\overset{N}{\underset{k=1}{\sum }}p_{k}\ln p_{k}$$

where 𝑁 is the optimal number of bins in the histogram. *N* can be determined using Equation (5):


(5) $$N=\exp\left[0.626+0.4\ln\left(M-1\right)\right]$$

*M* denotes the number of samples. The synchronization index $\rho_{n,m}$ was then computed as described in Equation (6):


(6) $$\rho_{n,m}=\frac{S_{\text{max}}-S}{S_{\text{max}}}$$

where the maximum possible entropy $S_{\text{max}}$ for a uniform distribution is calculated as $S_{\text{max}}=\ln N$ . By following these steps, we were able to quantify the degree of synchronization between heartbeat and stimuli during sleep. The synchronization index $\rho_{1,1}$ provides a measure of phase locking, with values ranging from 0 (no synchronization) to 1 (perfect synchronization).

### 2.5 Electroencephalography analysis

To evaluate the effects of the CLVS on cortical brain activity during sleep, EEG spectral analysis was performed at each sleep stage. Six-channel EEG signals (F3, F4, C3, C4, O1, and O2) were referenced to the contralateral mastoid region. EEG preprocessing consisted of resampling to 200 Hz and filtering with a 0.5–100 Hz band-pass filter. Before analyzing the EEG data, we detected the motion artifact segments based on the mean of the six-channel signals, which were normalized using the z-score method. The detected artifact segments were excluded from the analysis. Subsequently, we calculated the power spectral densities in the frequency bands of interest using the MATLAB pwelch function with a Hanning window of 4 s with 50% overlap from the EEG data: delta (0.5–4 Hz), theta (4–8 Hz), alpha (8–12 Hz), sigma (12–16 Hz), and beta (16–30 Hz). To account for individual variability, the power spectra were normalized by dividing the cumulative power by 30 Hz. The F3 EEG channel was used for analysis. If motion artifacts were found in >30% of the data from this channel, the opposite hemispherical channel data (i.e., the F4 EEG channel) were used for the analysis (Yook et al., 2024).

### 2.6 Coherence analysis

**Figure 4:**
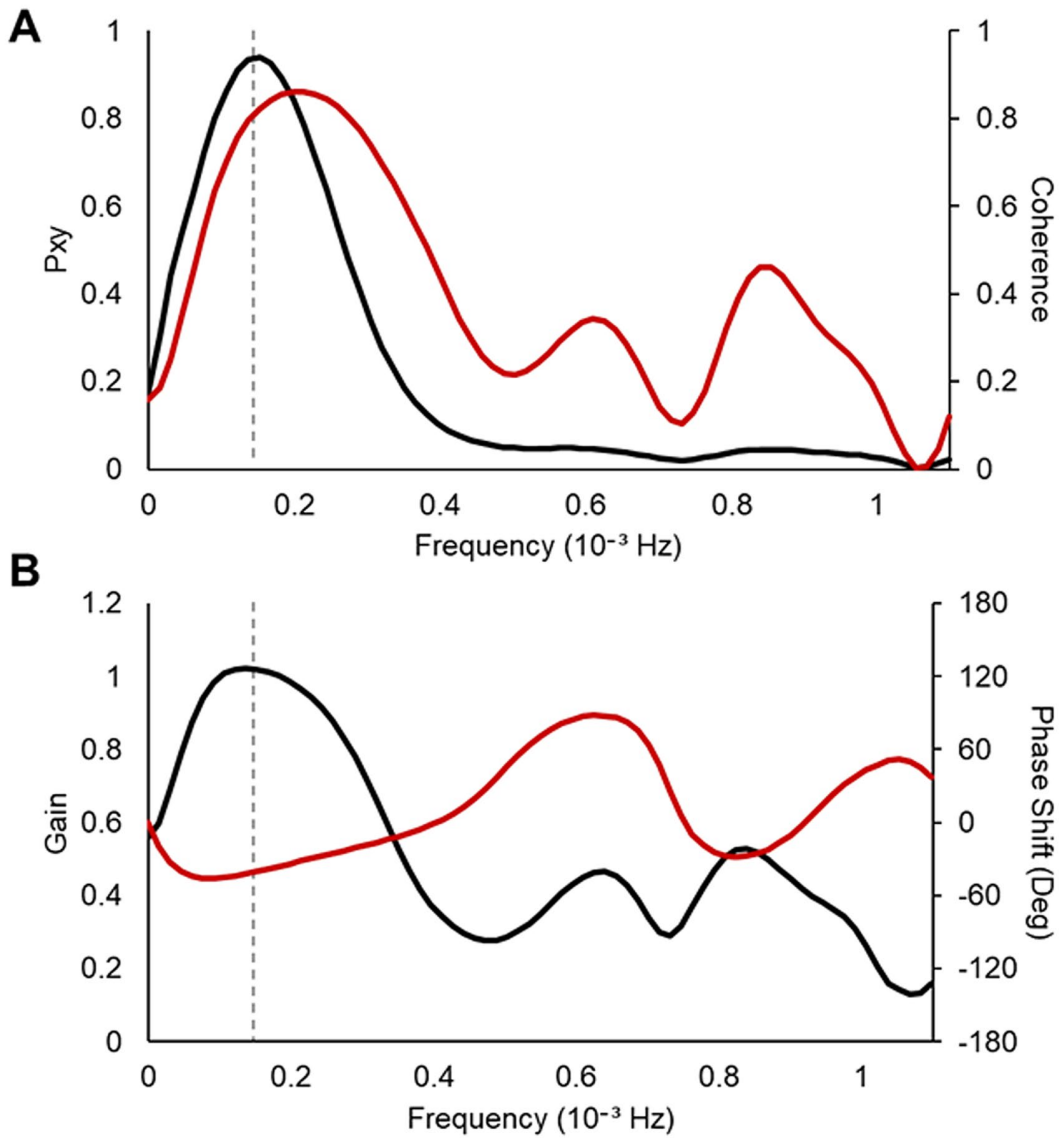
An example of the result for coherence analysis between the nHF–HRV and the normalized delta EEG power. **(A)** Values of the cross-spectrum $P_{xy}\left(f\right)$ (left axis, black line) and the coherence $C_{xy}\left(f\right)$ (right axis, red line) **(B)** Values of the gain (left axis, black line) and the phase shift in degree (right axis, red line). The frequency of the maximum of the cross-spectrum between the two signals is marked as gray dashed line.

To verify the effect of vibratory stimulation on the interaction between cardiac vagal and slow-wave neural activities, coherence analysis was applied to the nHF of the HRV indices and normalized delta EEG power (Jurysta et al., 2003). For coherence analysis, the data range was limited to the first three non-REM-REM cycles to ensure stationarity of the time series and high-frequency resolution of the power spectrum (Jurysta et al., 2009). An example of the coherence analysis is shown in Figure 4. We examined the frequency at which cross-spectrum $P_{xy}\left(f\right)$ reached its maximum value. At this frequency, we calculated the coherence $C_{xy}\left(f\right)$ , gain, and phase shift for comparison on SHAM and STIM nights (gray dashed line in Figure 4). The coherence value of 0 indicates total linear independence, whereas a value of 1 indicates complete linear dependence at a given frequency 𝑓.

The HRV, synchronization, EEG, and coherence analyses were performed using MATLAB (version R2023b; MathWorks, Natick, MA, USA).

### 2.7 Statistical analyses

To verify the effects of vibration stimulation on sleep, all extracted variables, such as the questionnaire, sleep parameters, HRV, synchronization index, relative EEG power, and coherence from the SHAM and STIM nights, were first tested for normality using the Shapiro–Wilk test with a significance level of *p* < 0.05. If the data met the criteria for a normal distribution, a paired t-test was performed; otherwise, the Wilcoxon signed-rank sum test was conducted. A *p* < 0.05 was considered significant.

In addition, correlations between the synchronization index and the extracted variables were analyzed to infer the associations between heart rate synchronization and sleep enhancement. If the variables were normally distributed, the Pearson correlation coefficient was computed; otherwise, Spearman’s rho was computed. All analyses were performed using the SPSS statistics program (version 25.0; SPSS Inc., Chicago, IL, USA).

## 3 Results

### 3.1 Questionnaire

Table 1 shows the responses to the questionnaire regarding the quality of sleep and discomfort caused by vibration stimulation after the completion of sleep on the SHAM and STIM nights. According to the responses to Q1 and Q2, there was no statistical difference in the time it took for participants to fall asleep, but the quality of sleep significantly increased (mean ± SD: SHAM, 2.3 ± 1.2; STIM, 3.1 ± 1.3; *p* = 0.033), with participants reporting better sleep during the night with vibration stimulation. The responses to Q3 indicated no difference in the perception of vibration stimulation during sleep.

**Table 1:** Questions about sleep and comfort of the stimulation (Mean ± SD).

Question	SHAM	STIM	*p*-value
Q1. Subjective sleep onset latency (min)	33.2 ± 35.4	47.3 ± 78.4	0.797
Q2. How was the sleep quality? (0–5, No sleep at all–Very good sleep)	2.3 ± 1.2	3.1 ± 1.3^*^	0.033
Q3. Felt external stimuli while sleeping (0–5, felt nothing–felt very well)	1.7 ± 1.7	1.7 ± 1.6	1.000
Q4. Discomfort due to the stimulus (0–5, No–Yes)	1.7 ± 1.7	1.2 ± 1.5	0.090

^*^p < 0.05.

### 3.2 Sleep macrostructure

We compared the sleep architecture parameters between SHAM and STIM nights to determine the effect of the CLVS on sleep structure (Table 2). All sleep stage percentages were calculated based on the sleep period time, and the percentages of sleep stages excluding WASO were calculated based on the total sleep time. A significant difference between SHAM and STIM was observed only for WASO. The total time spent in WASO and percentage of WASO under STIM were significantly lower compared with those under SHAM (mean ± SD: 37.0 ± 27.3 min and 9.1 ± 6.6% for STIM, 48.6 ± 30.3 min and 11.8 ± 7.6% for SHAM, *p* = 0.044). Moreover, the average duration of the WASO under STIM was significantly shorter than that under the SHAM (mean ± SD: STIM, 2.6 ± 1.5 min; SHAM, 3.2 ± 1.9 min; *p* = 0.031). Vibration stimulation did not affect the average duration, proportion, or length of N3 stage.

**Table 2:** Polysomnographic results in each experimental condition for the entire night (Mean ± SD).

Parameters		SHAM	STIM	*p*-value
TRT (min)		421.2 ± 3.1	417.7 ± 12.8	0.333
TST (min)		365.8 ± 33.9	371.6 ± 30.1	0.368
SPT (min)		414.4 ± 5.8	408.7 ± 14.2	0.147
SE (%)		86.9 ± 8.1	89.0 ± 6.8	0.398
SOL (min)		6.8 ± 5.5	9.1 ± 9.5	0.286
DEEPL (min)		57.9 ± 82.5	53.4 ± 37.7	0.749
REML (min)		131.9 ± 87.2	113.1 ± 48.3	0.463
AHI (event/h)		8.2 ± 6.6	9.4 ± 7.7	0.184
PLMI (event/h)		0.3 ± 1.1	0.6 ± 1.9	0.188
WASO	Total time (min)	48.6 ± 30.3	37.0 ± 27.3^*^	0.044
	Proportion (%SPT)	11.8 ± 7.6	9.1 ± 6.6^*^	0.050
	Average duration (min)	3.2 ± 1.9	2.6 ± 1.5^*^	0.031
Stage N1	Total time (min)	52.7 ± 25.3	51.0 ± 30.8	0.376
	Proportion (%TST)	14.6 ± 7.1	13.9 ± 8.4	0.355
	Proportion (%SPT)	12.7 ± 6.0	12.5 ± 7.6	0.494
	Average duration (min)	2.0 ± 0.5	2.0 ± 1.0	0.198
Stage N2	Total time (min)	227.4 ± 32.2	233.4 ± 31.2	0.313
	Proportion (%TST)	62.1 ± 6.2	62.8 ± 6.9	0.646
	Proportion (%SPT)	54.6 ± 7.5	57.1 ± 7.5	0.113
	Average duration (min)	9.1 ± 4.3	8.9 ± 3.3	0.748
Stage N3	Total time (min)	52.7 ± 25.3	51.0 ± 30.8	0.376
	Proportion (%TST)	14.6 ± 7.1	13.9 ± 8.4	0.355
	Proportion (%SPT)	4.9 ± 5.4	4.5 ± 5.0	0.508
	Average duration (min)	3.0 ± 3.1	3.3 ± 3.7	0.605
REM	Total time (min)	65.4 ± 22.7	68.9 ± 25.1	0.619
	Proportion (%TST)	17.9 ± 5.9	18.4 ± 5.9	0.772
	Proportion (%SPT)	15.8 ± 5.5	16.8 ± 5.9	0.540
	Average duration (min)	23.0 ± 19.1	20.5 ± 9.3	0.872

TRT, total recording time; TST, total sleep time; SPT, sleep period time; SE, sleep efficiency; SOL, sleep onset latency; DEEPL, deep sleep latency; REML, rapid eye movement sleep latency; AHI, apnea hypopnea index; PLMI, periodic limb movement index; WASO, wake after sleep onset; REM, rapid eye movement sleep; SHAM, condition without stimulation; and STIM, condition with stimulation. ^*^p < 0.05.

### 3.3 HRV and synchronization

**Figure 5:**
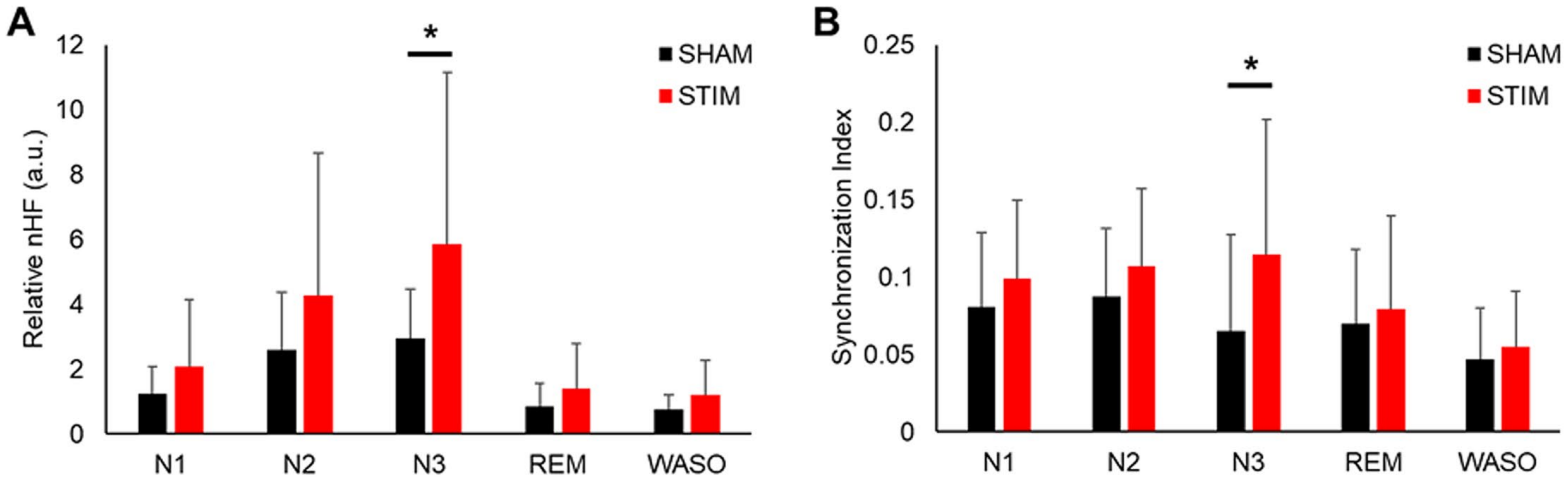
Effect of vibratory stimulation on heart rhythm in each sleep stage. **(A)** Mean ± SD of nHF index during the SHAM and STIM **(B)** Mean ± SD of synchronization index between heartbeat and stimuli during the SHAM and STIM. nHF, normalized low-frequency band power; N1, N2 and N3, non-REM sleep stages 1–3; REM, rapid eye movement sleep; WASO, wake after sleep onset. ^*^p < 0.05.

To investigate the effects of vibration stimulation on autonomic nervous activity during sleep, HRV and synchronization analyses were performed. The HRV parameters in both time and frequency domains calculated for each sleep stage are shown in Figure 5 and Supplementary Table S1. Among the HRV parameters, the nHF under STIM was significantly higher in the N3 sleep stage compared with that under SHAM (Figure 5A, mean ± SD: SHAM, 2.94 ± 1.52; STIM, 5.85 ± 5.31; *p* = 0.046). Except for this case, there were no significant differences in the HRV parameters between the STIM and SHAM nights.

The synchronization analysis for each sleep stage revealed that the synchronization index between the heartbeats and stimuli in the N3 sleep stage under STIM was significantly higher than that under SHAM (Figure 5B, mean ± SD: SHAM, 0.07 ± 0.06; STIM, 0.11 ± 0.09; *p* = 0.033). There were no significant changes in the other sleep stages.

### 3.4 Relative EEG power

**Figure 6:**
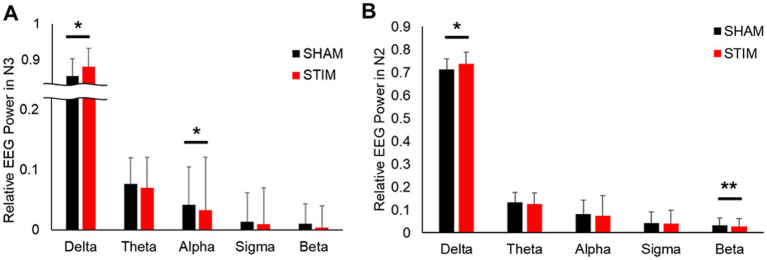
Effect of vibratory stimulation on relative EEG power during **(A)** Non-REM sleep stage 3 and **(B)** Non-REM sleep stage 2 and 3. ^*^p < 0.05, ^**^p < 0.01.

To assess the effects of CLVS on cortical brain activity, spectral analysis of EEG signals was performed (Figure 6 and Supplementary Table S2). The results showed that the relative delta frequency band EEG power under STIM was significantly higher in the N2 sleep stage and N3 sleep stage compared with SHAM (mean ± SD: N2 sleep stage, SHAM, 0.713 ± 0.068; STIM, 0.737 ± 0.054; *p* = 0.049; N3 sleep stage, SHAM, 0.856 ± 0.049; STIM, 0.881 ± 0.053; *p* = 0.049). In the N2 sleep stage, the beta frequency band relative power was significantly lower under STIM night compared with under SHAM night (mean ± SD: SHAM, 0.031 ± 0.030; STIM, 0.026 ± 0.023; *p* < 0.01), and in the N3 sleep stage, a significant decrease in the alpha frequency band relative power under STIM was observed (mean ± SD: SHAM, 0.042 ± 0.020; STIM, 0.034 ± 0.016; *p* = 0.025).

### 3.5 Coherence between HRV and EEG

The results of the coherence analysis between the nHF and normalized delta EEG power are summarized in Table 3. The coherence between both signals was significantly larger on STIM night compared with SHAM night (mean ± SD: SHAM, 0.61 ± 0.17; STIM, 0.72 ± 0.17; *p* = 0.039). The gain was generally higher on STIM night than on SHAM night, but the difference was not statistically significant (mean ± SD: SHAM, 0.80 ± 0.21; STIM, 0.89 ± 0.17; *p* = 0.112). There were no significant differences in the phase shift, expressed in degrees or minutes, between the SHAM and STIM groups. A negative phase shift indicates that cardiac vagal activity leads to slow-wave neural activity in the phase.

**Table 3:** Results of coherence analysis in each experimental condition for the entire night (Mean ± SD).

	SHAM	STIM	*p*-value
Coherence	0.61 ± 0.17	0.72 ± 0.17^*^	0.039
Gain	0.80 ± 0.21	0.89 ± 0.17	0.112
Phase Shift (deg)	−10.05 ± 8.33	−15.66 ± 18.79	0.809
Phase Shift (min)	−3.05 ± 2.69	−5.26 ± 5.89	0.687

^*^p < 0.05.

### 3.6 Correlation analyses

**Figure 7:**
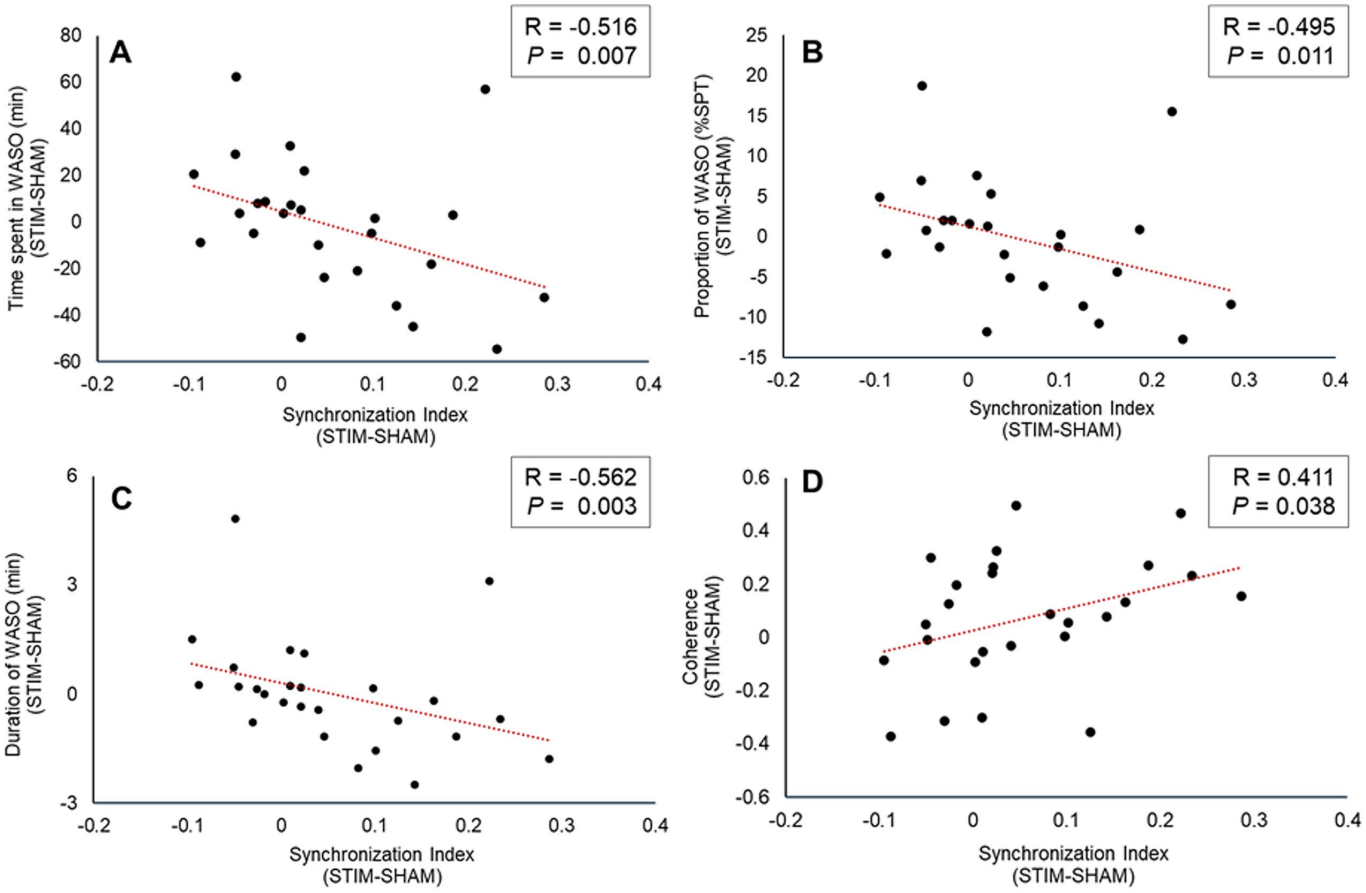
Scatter plot between the change (STIM minus SHAM) of the synchronization index during N3 sleep and sleep variables **(A)** change of total time spent in WASO **(B)** change of proportion of WASO **(C)** change of total time spent in WASO **(D)** change of coherence between nHF parameter and relative delta frequency band EEG power. WASO, wake after sleep onset; SPT, sleep period time; SHAM, condition without stimulation; and STIM, condition with stimulation.

We performed a correlation analysis between the change in the synchronization index of N3 sleep (STIM–SHAM) and the WASO-related variables that showed significant differences between the SHAM and STIM conditions among the PSG-derived sleep parameters. As shown in Figure 7, the change in the synchronization index exhibited a significant negative correlation with WASO time, percentage, and average duration (total time spent in WASO: correlation coefficient = −0.516, *p* = 0.007, Figure 7A; proportion of WASO: correlation coefficient = −0.495, *p* = 0.011, Figure 7B; Average duration of WASO: correlation coefficient = −0.562, *p* = 0.003, Figure 7C). In addition, we analyzed the correlation between changes in the synchronization index of N3 sleep and coherence. The correlation between the change in coherence and the synchronization index was positive and statistically significant (correlation coefficient = 0.411, *p* = 0.038, Figure 7D).

## 4 Discussion

This study examined the effects of CLVS on sleep quality and cardiac autonomic regulation in adults with poor sleep quality. In this study, we applied a smart mattress equipped with a heart rate sensor and woofer for vibration stimulation to the nocturnal sleep of 27 participants to compare sleep with and without CLVS.

### 4.1 Effect of CLVS on subjective and objective sleep quality

The responses to Q3 in Table 1 indicate that the participants could not distinguish between SHAM and STIM nights, suggesting that the vibrations were delivered at an imperceptible level. Interestingly, discomfort due to the stimulus was marginally higher during the SHAM night compared with the STIM night (mean ± SD: Q4, SHAM = 1.7 ± 1.7, STIM = 1.2 ± 1.5, *p* = 0.090), suggesting that the vibration stimulation was not disruptive and the participants originally experienced sleep disturbances. This is supported by the lower average sleep efficiency (SE) and WASO in our study compared with the previous study by Choi et al. (2021), which involved healthy participants (mean ± SD; Choi et al., 2021, 91.3 ± 4.8% and 28.17 ± 17.83 min; our study, 86.9 ± 8.1% and 48.6 ± 30.3 min; Table 2). Similar to a previous study, there was no significant difference in SOL between the SHAM and STIM nights. However, at STIM night, the total time, proportion, and average duration of WASO significantly decreased (Table 2), leading to statistically significant improvements in both subjective and objective sleep quality (Zitser et al., 2022), supporting our hypothesis that CLVS has a sleep-enhancing effect on poor sleep quality. Although WASO was significantly reduced in this study, it was still longer than that of normal sleepers in a previous study (Choi et al., 2021). There remains room for future advancement in the method to reduce the WASO of poor sleepers to approach that of normal sleepers.

This study includes a mild (*N* = 8) and a moderate (*N* = 5) sleep apnea group, and apneic event may affect the signal quality of non-invasive sensors and sleep quality (Kania et al., 2022; Kwon et al., 2022). Therefore, we evaluated the effects of the condition (SHAM and STIM nights) and sleep apnea (AHI < 5 and AHI ≥ 5) on total time of WASO using a two-way repeated measures ANOVA. The results showed a significant main effect for the condition (*F* = 4.370, *p* = 0.045) but not for sleep apnea (*F* = 1.703, *p* = 0.201). The interaction between the two factors (condition and sleep apnea) was also not significant (*F* = 0.001, *p* = 0.973). In sum, sleep apnea did not affect the WASO-reducing effect of CLVS.

### 4.2 Effect of CLVS on autonomic nervous and cortical brain activity during sleep

Among the HRV indices, nHF is mainly a marker of cardiac parasympathetic modulation (Pini et al., 2022; Yoon et al., 2018). Previous studies have identified ANS dysregulation, with a predominance of sympathetic activation and diminished slow-wave EEG activity, as significant factors in the relationship between poor sleep quality and sleep disorders (Krystal et al., 2002; Hall et al., 2007; Oliveira-Silva et al., 2020). In agreement with previous studies (Choi et al., 2019; Choi et al., 2021), the nHF was significantly increased during N3 sleep, and the synchronization index between an individual’s heartbeat and vibration stimuli also showed a significant increase during N3 sleep under STIM conditions (Figures 5A,B). Moreover, we observed negative correlations between changes (STIM–SHAM) in the synchronization index during N3 sleep and changes in WASO-related variables (Figures 7A–C). This indicates that vibration stimulation has a beneficial effect on sleep quality by enhancing the resting function of the parasympathetic nervous system and increasing sleep depth.

Our results showed that the relative delta frequency band EEG power during N2 and N3 sleep significantly increased under STIM compared with other sleep stages (Figures 6A,B), replicating prior observations (Choi et al., 2021). In particular, we observed a significant decrease in alpha power during N3 sleep and a significant decrease in beta power during N2 sleep simultaneously. To determine whether these reciprocal changes occur simultaneously or independently, we further compared the delta/alpha ratio (DAR) during N3 sleep and the delta/beta ratio (DBR) during N2 sleep. In our results, both DAR and DBR showed significant increases (mean ± SD: DAR, SHAM = 27.18 ± 16.19, STIM = 39.29 ± 44.52, *p* = 0.032; DBR, SHAM = 31.25 ± 13.50, STIM = 35.03 ± 11.46, *p* = 0.013), indicating that sleep depth was higher during the STIM nights in both N2 and N3 stages. Hyperarousal, characterized by the abnormal activation of the central and peripheral nervous systems that disrupts the onset and continuity of sleep, is considered a key component of insomnia disorder (Riedner et al., 2016). A substantial amount of research focusing on the neurocognitive interpretation of hyperarousal through spectral analysis of sleep microstructure in patients with insomnia disorder has shown a significant association with lower delta EEG activity and greater alpha, sigma, and beta activity during non-REM sleep compared to healthy controls (Krystal et al., 2002; Zhao et al., 2021). Specifically, Riedner et al. (2016) observed significantly higher alpha EEG activity in the N3 sleep in patients with insomnia disorder compared to normal subjects. Given our findings of reduced WASO along with significant increases in DBR during N2 sleep and DAR during N3 sleep, it suggests that CLVS may effectively mitigate hyperarousal states that contribute to sleep disturbances.

Recent studies have assessed bidirectional interaction between the brain and heart across different sleep stages (Arakaki et al., 2023). Lechinger et al. (2015) showed through heartbeat-evoked potentials during N3 sleep that cardiac activity can modulate or be modulated by ongoing oscillatory brain activity. Considering the possibility that cardiac activity can influence brain function (Grimaldi et al., 2019) and our finding that the nHF phase leads to delta waves in coherence analysis, it is plausible that autonomic modulation induced by vibration stimulation increases slow-wave EEG power during N2 and N3 sleep. However, the current study was not designed to elucidate the heart–brain relationship mediating the effects of vibration stimulation on sleep quality, and the results were mostly obtained through correlation analysis. Therefore, functional causality and directionality analyses of the heart–brain–sleep connection are required for physiological interpretation.

Disrupted sleep affects CNS–ANS coupling (De Zambotti et al., 2018; Jurysta et al., 2009; Jurysta et al., 2013). Jurysta et al. (2009) performed a coherence analysis between nHF and relative delta frequency EEG power in the sleep of patients with insomnia and reported decreased linear coupling and a more variable phase shift with respect to controls. Our results showed significantly increased coherence between the nHF and delta EEG in the STIM with CLVS group compared with the SHAM group (Table 3). In addition, coherence positively correlated with the synchronization index during N3 sleep. This implies that CLVS increases the magnitude of cardiac autonomic activity and the delta frequency of EEG and, beyond that, strengthens their coupling by modulating cardiac activity synchronized with vibratory stimuli. However, while the coherence significantly increased in the STIM compared to the SHAM, gain did not reach statistical significance, although it did increase on average (mean ± SD: SHAM = 0.80 ± 0.21, STIM = 0.89 ± 0.17, *p* = 0.112). This lack of significance in gain might be attributed to insufficient data.

### 4.3 Limitation and future work

The current study has some limitations. First, we used PSQI scores to classify poor sleep quality rather than clinically diagnosis of insomnia. Although the PSQI cut-off of >5 has a high sensitivity of 98.7% and a high specificity of 84.4% for distinguishing between insomnia patients and healthy controls (Backhaus et al., 2002), the exclusion of clinical evaluation means that our findings may not fully generalize to the insomnia population. Therefore, future studies should apply the same methodology to individuals who have been clinically diagnosed with insomnia. Second, although we focused on improving sleep structure as the primary measure of CLVS effectiveness, the effect of CLVS on related conditions, such as mood, anxiety, and attention, remains unclear. As poor sleep quality is closely associated with such conditions (Yo et al., 2022), future studies should explore these aspects in detail. Third, although unconstrained real-time heartbeat detection and feedback mechanism of the smart mattress-type CLVS system represents a significant advancement, our validation was limited to a laboratory environment using standard PSG. A previous study indicated that sleep posture and habits, which might affect signal quality, significantly differed between laboratory and home settings (Correia et al., 2023). Further validation in home settings is necessary to ensure practical use. Finally, the current investigation was limited to a single night’s sleep using PSG recordings. Although the results were promising, it is crucial to conduct further studies to evaluate the sustained effect of CLVS over extended periods, as long-term use may reveal additional benefits or potential drawbacks that are not evident in short-term studies.

## 5 Conclusion

This study investigated the effects of CLVS on sleep quality, nocturnal cardiac autonomic regulation, and cortical brain activity in adults with sleep disturbance. The results showed improvements in both the subjective and objective measures of sleep quality. Vibration stimulation was associated with increased nocturnal HRV indices of vagal activity, slow-wave activity, and coherence between these measures, all of which were associated with enhanced sleep quality. Considering that the reduction in WASO was significant only for poor sleepers, CLVS is expected to be more effective for sleepers with sleep disturbances. Given its higher practical applicability compared with other noninvasive methods, CLVS can provide a novel approach for enhancing sleep quality and serve as a non-pharmacological alternative for elderly populations and individuals whose conditions are closely associated with sleep problems.

## Data Availability

The datasets presented in this article are not readily available because concerns regarding participant/patient anonymity. Requests to access the datasets should be directed to the corresponding author.
