# Supplementary material for: Effect of closed-loop vibration stimulation on sleep quality for poor sleepers
Source: Front Neurosci. 2024 Oct 7;18:1456237. doi: 10.3389/fnins.2024.1456237 (PMC11491432; doi:10.3389/fnins.2024.1456237)
Supplement: Supplementary file 1 [file Data_Sheet_1.DOCX]

Supplementary Material

# Supplementary Figures and Tables

## Supplementary Figures


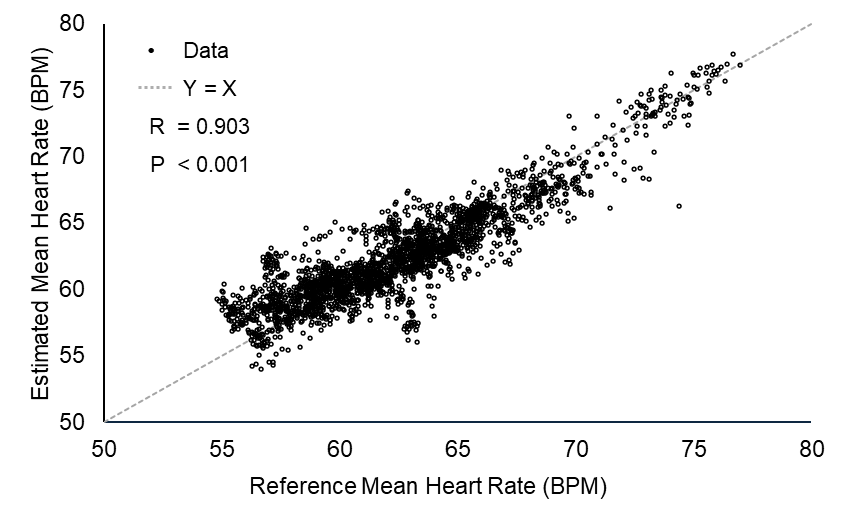


**Supplementary Figure 1.** The correlation result between the estimated mean 5-minute heart rate and the reference mean 5-minute heart rate. The estimated heart rate was computed from signals collected by the piezoelectric film sensor embedded in the smart mattress and processed by a microprocessor, while the reference heart rate was calculated from R-R intervals in the ECG signals in PSG recording (number of samples = 2436, Pearson's correlation coefficient = 0.903, *p*-value < 0.001, mean absolute percentage error = 2.139%).

## Supplementary Tables

**Supplementary Table 1.** HRV parameters during SHAM/STIM conditions and sleep stages (Mean ± SD)

| stages | condition | mHR | SDNN | nLF | nHF | HFLF | TF | SI |
| --- | --- | --- | --- | --- | --- | --- | --- | --- |
| N1 | SHAM | 0.96 ± 0.05 | 1.85 ± 0.68 | 1.15 ± 1.05 | 1.22 ± 0.87 | 1.75 ± 1.76 | 1.00 ± 0.06 | 0.08 ± 0.05 |
|  | STIM | 0.97 ± 0.04 | 1.75 ± 0.53 | 1.16 ± 0.91 | 2.07 ± 2.10 | 2.99 ± 3.69 | 1.03 ± 0.23 | 0.10 ± 0.05 |
| N2 | SHAM | 0.94 ± 0.05 | 1.47 ± 0.50 | 1.33 ± 1.15 | 2.58 ± 1.79 | 3.46 ± 3.08 | 0.98 ± 0.07 | 0.09 ± 0.04 |
|  | STIM | 0.96 ± 0.05 | 1.45 ± 0.44 | 1.20 ± 0.98 | 4.29 ± 4.40 | 6.82 ± 8.70 | 1.02 ± 0.22 | 0.11 ± 0.05 |
| N3 | SHAM | 0.96 ± 0.05 | 1.16 ± 0.39 | 1.13 ± 0.97 | **2.94 ± 1.52** | 4.98 ± 4.60 | 0.96 ± 0.07 | **0.07 ± 0.06** |
|  | STIM | 0.96 ± 0.06 | 1.24 ± 0.52 | 1.03 ± 0.86 | **5.85 ± 5.31^*^** | 11.07 ± 12.31 | 1.02 ± 0.25 | **0.11 ± 0.09^*^** |
| REM | SHAM | 0.99 ± 0.08 | 1.53 ± 0.66 | 0.93 ± 0.79 | 0.86 ± 0.69 | 1.47 ± 1.18 | 1.00 ± 0.07 | 0.07 ± 0.05 |
|  | STIM | 1.01 ± 0.07 | 1.63 ± 0.75 | 0.94 ± 0.90 | 1.39 ± 1.39 | 2.53 ± 2.44 | 1.05 ± 0.25 | 0.08 ± 0.06 |
| WASO | SHAM | 1.04 ± 0.07 | 2.30 ± 0.87 | 0.79 ± 0.63 | 0.74 ± 0.48 | 1.47 ± 1.43 | 1.02 ± 0.06 | 0.05 ± 0.03 |
|  | STIM | 1.07 ± 0.07 | 2.32 ± 0.76 | 0.84 ± 0.62 | 1.19 ± 1.08 | 2.21 ± 2.36 | 1.06 ± 0.24 | 0.05 ± 0.04 |

N1, N2 and N3; Non-REM sleep stages 1-3; REM, rapid eye movement sleep; WASO, wake after sleep onset; mHR, mean heart rate; SDNN, standard deviation of the RR intervals; nLF, normalized low-frequency band power; nHF, normalized high-frequency band power; and HFLF, ratio of the high-frequency power to low-frequency power; TF, total frequency power; and SI, synchronization index. ^*^ *p*-value < 0.05.

**Supplementary Table 2.** EEG relative power values during SHAM/STIM conditions and sleep stages (Mean ± SD)

| stages | condition | Delta | Theta | Alpha | Sigma | Beta |
| --- | --- | --- | --- | --- | --- | --- |
| N1 | SHAM | 0.615 ± 0.063 | 0.159 ± 0.027 | 0.104 ± 0.023 | 0.048 ± 0.014 | 0.075 ± 0.036 |
|  | STIM | 0.636 ± 0.055 | 0.153 ± 0.029 | 0.098 ± 0.029 | 0.045 ± 0.011 | 0.068 ± 0.030 |
| N2 | SHAM | **0.713 ± 0.068** | 0.133 ± 0.022 | 0.081 ± 0.024 | 0.043 ± 0.019 | **0.031 ± 0.030** |
|  | STIM | **0.737 ± 0.054^*^** | 0.125 ± 0.030 | 0.073 ± 0.024 | 0.039 ± 0.013 | **0.026 ± 0.023^*^** |
| N3 | SHAM | **0.856 ± 0.049** | 0.077 ± 0.029 | **0.042 ± 0.020** | 0.014 ± 0.012 | 0.011 ± 0.019 |
|  | STIM | **0.881 ± 0.053^*^** | 0.071 ± 0.035 | **0.034 ± 0.016^*^** | 0.010 ± 0.006 | 0.005 ± 0.002 |
| REM | SHAM | 0.611 ± 0.062 | 0.178 ± 0.024 | 0.094 ± 0.026 | 0.038 ± 0.007 | 0.078 ± 0.034 |
|  | STIM | 0.620 ± 0.072 | 0.179 ± 0.028 | 0.099 ± 0.036 | 0.038 ± 0.010 | 0.064 ± 0.018 |
| WASO | SHAM | 0.531 ± 0.113 | 0.129 ± 0.030 | 0.191 ± 0.109 | 0.048 ± 0.015 | 0.101 ± 0.036 |
|  | STIM | 0.543 ± 0.129 | 0.115 ± 0.033 | 0.202 ± 0.134 | 0.045 ± 0.011 | 0.095 ± 0.026 |

N1, N2 and N3; Non-REM sleep stages 1-3; REM, rapid eye movement sleep; and WASO, wake after sleep onset. * p-value < 0.05.
